# Regulatory 3′ Untranslated Regions of Bacterial mRNAs

**DOI:** 10.3389/fmicb.2017.01276

**Published:** 2017-07-10

**Authors:** Gai-Xian Ren, Xiao-Peng Guo, Yi-Cheng Sun

**Affiliations:** MOH Key Laboratory of Systems Biology of Pathogens, Institute of Pathogen Biology, Chinese Academy of Medical Sciences and Peking Union Medical CollegeBeijing, China

**Keywords:** 3′untranslated region, mRNA decay, mRNA translation initiation, bacteria, post-transcriptional regulation

## Abstract

The untranslated regions (UTRs) of mRNA contain important features that are relevant to the post-transcriptional and translational regulation of gene expression. Most studies of bacterial UTRs have focused on the 5′regions; however, 3′UTRs have recently emerged as a new class of post-transcriptional regulatory elements. 3′UTRs were found to regulate the decay and translation initiation in their own mRNAs. In addition, 3′UTRs constitute a rich reservoir of small regulatory RNAs, regulating target gene expression. In the current review, we describe several recently discovered examples of bacterial regulatory 3′UTRs, discuss their modes of action, and illustrate how they facilitate gene regulation in various environments.

## Introduction

Environmental conditions experienced by free-living organisms, such as nutrient availability, temperature, oxygen, pH, and osmolarity, frequently change. To adapt to the ever-changing environment, bacteria have established an intricate network of regulators to accurately modulate gene expression. Regulation of gene expression in prokaryotes takes place primarily at the transcriptional level, i.e., by the activation or repression of transcription; however, post-transcriptional regulation of gene expression may play a critical role when bacteria must rapidly adjust to the changing environment.

mRNA functions primarily as a carrier of genetic information; however, its sequence, especially the 5′ and 3′untranslated regions (UTRs), contains many features that can modulate gene expression at the post-transcriptional level (Pesole et al., 2001). During the last few decades, significant progress has been made in the understanding of eukaryotic and prokaryotic 5′UTR-mediated gene regulation (Chen et al., 1991; Sonenberg, 1994; Agaisse and Lereclus, 1996; Oliva et al., 2015; Hinnebusch et al., 2016). RNA thermometers and riboswitches have been found in 5′UTRs and have been extensively studied in bacteria; these elements regulate gene in both transcriptional and translational levels, as well as mRNA stability (Henkin, 2008; Breaker, 2011; Kortmann and Narberhaus, 2012; Serganov and Patel, 2012; Krajewski and Narberhaus, 2014). In addition, RNA-binding proteins bind to 5′UTRs to regulate gene expression by modulating the accessibility of ribosome-binding sites (RBSs) on mRNAs (Babitzke et al., 2009; Van Assche et al., 2015).

**FIGURE 1:**
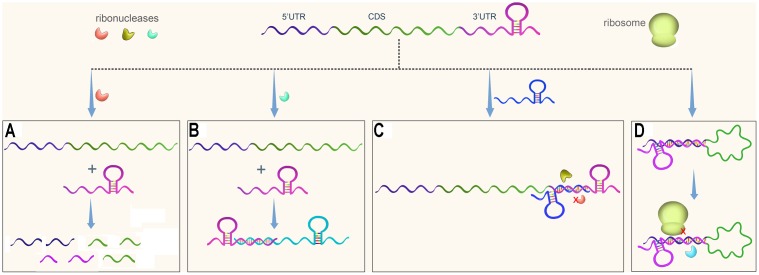
Regulatory pathways of 3′UTRs in bacteria. An mRNA usually contains three regions, the 5′UTR, the coding sequence (CDS), and the 3′UTR. **(A)** A 3′UTR is recognized and cleaved by ribonuclease to initiate mRNA decay. **(B)** A 3′UTR-derived sRNA regulates expression of its target gene. **(C)** A 3′UTR functions as the target of regulatory sRNA, resulting in protection of the 3′UTR from degradation or promoting its degradation. **(D)** A 3′ UTR interacts with the 5′ region of its own gene, potentially affecting mRNA stability and translational initiation.

In eukaryotes, 3′UTRs regulate gene transcription by modulating mRNA decay, translation, or localization, and these processes have been well studied (St Johnston, 1995; Pesole et al., 2001; Wilkie et al., 2003; Barreau et al., 2005). It was traditionally believed, however, that bacterial 3′UTRs mainly contain transcriptional terminators, which are either Rho-dependent or Rho-independent. Recently, 3′UTRs were found to be involved in post-transcriptional gene regulation in bacteria as well (**Figure 1**). 3′UTRs especially long 3′UTRs can be cleaved by ribonuclease to initiate mRNA decay (**Figure 1A**).

3′UTRs are a rich reservoir of small regulatory RNAs and regulate target gene expression (**Figure 1B**). In addition, 3′UTRs are a target of regulatory sRNAs (**Figure 1C**). Finally, 3′UTRs interact with 5′UTRs to regulate translation initiation (**Figure 1D**). This paper summarizes several papers published in the last several years that suggest that 3′UTRs function as new post-transcriptional regulators. The regulatory mechanisms of 3′UTRs and how these genetic elements facilitate gene regulation in response to the changing environment are discussed.

## 3′UTRs Undergo Ribonuclease Cleavage

Regulation of mRNA decay is an important mechanism of post-transcriptional control of gene expression. In bacteria, mRNA decay depends on the rate-determining initial step (Lalaouna et al., 2013; Laalami et al., 2014). Following an initial endonucleolytic cleavage, mRNA fragments are subsequently degraded by exoribonucleases. Ribosomes shield mRNA from degradation by endonucleolytic enzymes (Deneke et al., 2013). Hence, available (untranslated) mRNA regions are more likely to be attacked by ribonucleases than translated mRNA regions. Selinger et al. (2003) analyzed the positional patterns of transcript degradation in *Escherichia coli* using subgenic-resolution oligonucleotide microarrays; they found that, although 5′UTRs are generally less stable than 3′UTRs, some 3′UTRs were also unstable. This indicated that mRNA decay might be initiated from the 3′UTRs as well (Selinger et al., 2003). Consistent with these microarray data, 3′UTR-mediated mRNA decay was reported recently (Maeda and Wachi, 2012; Lopez-Garrido et al., 2014; Liu et al., 2016; Zhu et al., 2016).

3′UTRs may be attacked by an endonuclease, initiating mRNA decay. One early example of this was the *C. glutamicum aceA* 3′UTR (Maeda and Wachi, 2012). The *aceA* gene encodes isocitratelyase, which catalyzes the cleavage of isocitrate to succinate and glyoxylate (Gerstmeir et al., 2003). The *aceA* 3′UTR is involved in negative regulation of its own gene expression and the expression of *lacZ* fusion (Maeda and Wachi, 2012). Further analysis showed that the *aceA* 3′UTR contains a single-stranded AU-rich region, which might constitute the cleavage target of RNase E/G (Maeda and Wachi, 2012). RNase E/G- and 3′UTR-mediated degradation of *aceA* mRNA may enable instantaneous adjustment of cellular metabolism (Maeda and Wachi, 2012).

The *aceA* 3′UTR contains only 63 nt (Maeda and Wachi, 2012); however, the subsequently identified 3′UTRs that are involved in mRNA decay are usually longer. The 3′UTR of *hilD* mRNA (encoding a transcriptional regulator of *S. enterica* pathogenicity island 1) contains 310 nt (Lopez-Garrido et al., 2014). The presence of the *hilD* 3′UTR reduces the expression of its own gene and the *gfp* reporter gene, indicating that the *hilD* 3′UTR functions as an independent module regulating gene expression (Lopez-Garrido et al., 2014). Further analysis showed that the *hilD* 3′UTR is a target during *hilD* mRNA degradation by a degradosome containing RNase E and PNPase (Lopez-Garrido et al., 2014).

Another example of mediation of gene expression by a long 3′UTR is the *hmsT* 3′UTR, which contains 283 nt (Zhu et al., 2016). The *hmsT* gene encodes a diguanylate cyclase that stimulates biofilm formation in *Y. pestis* by synthesizing the secondary messenger c-di-GMP (Kirillina et al., 2004). The *hmsT* 3′UTR negatively modulates *hmsT* mRNA decay, in which PNPase is involved. This 3′UTR strongly represses gene expression at 37°C, but only weakly affects gene expression at 21°C, suggesting that the temperature is a signal that can be sensed by the *hmsT* 3′UTR to regulate its gene expression under changing environmental conditions (Zhu et al., 2016).

Both *hilD* and *aceA* possess a Rho-independent terminator (Maeda and Wachi, 2012; Lopez-Garrido et al., 2014), while *hmsT* has a Rho-dependent terminator (Zhu et al., 2016). Recently, it was reported that Rho-dependent termination is required for PNPase-mediated turnover of *slrA* mRNA in *Bacillus subtilis* (Liu et al., 2016). Replacement of the Rho-dependent terminator by a Rho-independent terminator in the *slrA* gene eliminated the PNPase-mediated turnover of mRNA, indicating that the *slrA* 3′UTR might also be involved in the regulation of mRNA decay. The 3′UTRs of *hilD* and *aceA*, containing Rho-independent terminators, possess a specific AU-rich regulatory region. The AU-rich region might be recognized and cleaved by endonuclease to initiate mRNA decay (Maeda and Wachi, 2012; Lopez-Garrido et al., 2014). However, the 3′UTRs of *hmsT* and *slrA*, containing Rho-dependent terminators, lack a specific regulatory region, and, in this situation, PNPase is primarily responsible for efficient mRNA turnover from the 3′ ends (Liu et al., 2016; Zhu et al., 2016).

There is a unique advantage to initiating transcript decay from the 3′UTRs rather than the 5′UTRs. Although initiating transcript decay from the 5′UTRs can rapidly inactivate a functional mRNA by removing the RBS, it cannot prevent translation that has already started from producing a full-length protein. Initiating the decay of a transcript from the 3′UTRs can rapidly halt translation by removing nearby encoding sequences. Hence, although 3′UTR-mediated mRNA decay is not an economical method of regulating gene expression, it might allow for a quicker response to the changing environment. Thus, mediation of mRNA turnover via the 3′UTRs might be useful for rapid control of gene expression in bacteria.

## 3′UTRs Interact with 5′UTRs

Translational control in eukaryotes is largely conferred by specific *cis*-acting sequences located in the 3′UTRs that serve as binding sites for the associated *trans*-acting factors (Sonenberg, 1994; Mazumder et al., 2003; Wilkie et al., 2003). By contrast, translational control in bacteria is mainly modulated through the 5′UTRs, which contains the Shine–Dalgarno (SD) sequence (Babitzke et al., 2009; Geissmann et al., 2009; Nakamoto, 2009; Waters and Storz, 2009). Recently, it was reported that the 3′UTR of *icaR* mRNA can interact with the 5′UTR to affect mRNA stability and translation in *Staphylococcus aureus* (Ruiz de los Mozos et al., 2013). A UCCCCUG motif is located at the 3′UTR of the *icaR* transcript, and is complementary to the SD region in the 5′UTR. On one hand, this interaction provides a double-stranded RNA substrate for RNase III cleavage to promote mRNA decay. On the other hand, it inhibits ribosome binding and hinders the formation of a translational complex, thus repressing the translation. This study illustrates that the bacterial 3′UTRs can interact with the SD region in the 5′UTRs of the same transcript leading to post-transcriptional regulation of gene expression (Ruiz de los Mozos et al., 2013). There are two additional examples for the modulation of bacterial translation by the interaction between 3′UTRs and 5′UTRs. It has been reported that the 3′ end of a full-length *hok* mRNA folds back to pair with its translational region to form a closed structure (Thisted et al., 1995). The formation of the closed structure renders the translation of the *hok* gene (Thisted et al., 1995). Another example is RNAIII (514 nt), the most studied regulatory RNA of *S. aureus*, which actually encodes a small peptide and contains a long 3′UTRs (354 nt) (Balaban and Novick, 1995; Felden et al., 2011). The RNAIII forms several regulatory structures including structures that facilitate interactions between the 5′ end and the 3′ end that regulate the expression of different target genes (Novick et al., 1993; Felden et al., 2011). Less is known about whether the formation of these structures regulates the translation of its own gene product. It has been reported that deletion of the 3′UTRs abolishes the temporal delay between transcription and translation of RNAIII (Balaban and Novick, 1995). The mechanism was not clarified, but it was proposed that the 3′UTRs might interact with the 5′UTRs to inhibit translation (Balaban and Novick, 1995).

## 3′UTRs that Function as sRNA Targets

It is reported that 3′UTRs might overlap with adjacent transcripts encoded on the opposite DNA strand (Rasmussen et al., 2009; Lasa et al., 2011). These overlapping 3′UTRs may be targeted by neighboring genes (usually encoding sRNAs) via a *cis*-acting antisense RNA mechanism. One example is a pair of toxin-antitoxin modules, TxpA and RatA (Silvaggi et al., 2005). RatA, an RNA antitoxin, is a sRNA (222 nt long) that inhibits the accumulation of mRNA of the toxic gene *txpA*. The orientations of *txpA* and *ratA* genes are convergent, and the genes overlap by ca. 75 nt; thus the 3′end of *txpA* mRNA is complementary to the sRNA *ratA*, thereby triggering *txpA* degradation (Silvaggi et al., 2005). Another example is the regulation of *gadX* by sRNA GadY. GadX is involved in the regulation of acid resistance in *E. coli* (Opdyke et al., 2004). The sRNA GadY overlaps with the 3′UTR of the *gadX* gene, and this overlap region is necessary for the regulation of *gadX* by *gadY*. Unlike other 3′UTRs, the interaction of the *gadX* 3′UTR with GadY increases the mRNA stability (Opdyke et al., 2004). The above examples illustrate mRNA crosstalk, whereby 3′UTRs act as sRNA targets to influence their own gene expression by positively or negatively modulating mRNA stability.

## 3′UTRs that Function as sRNA Reservoirs

sRNAs are usually associated with the RNA-binding protein Hfq in Gram negative bacteria, which facilities their regulatory function and protects sRNAs from degradation (De Lay et al., 2013; Wagner, 2013). Recently, 3′ regions of many mRNAs have been co-immunoprecipitated with Hfq, suggesting that the cellular functions of these 3′UTRs are independent of the role of the protein encoded by their parental mRNA, and might indeed function as sRNA reservoirs (Chao et al., 2012; Gossringer and Hartmann, 2012; Tree et al., 2014). CpxQ, an sRNA derived from the 3′ end of *cpxP* mRNA, is an excellent example of a 3′UTR acting as a sRNA reservoir (Chao and Vogel, 2016). CpxP, a periplasmic protein, combats envelope stress by tagging misfolded membrane proteins for degradation (Danese and Silhavy, 1998). The 3′UTR-derived sRNA CpxQ (60-nt-long) is generated during mRNA decay by RNase E and functions as an Hfq-dependent repressor of multiple mRNAs encoding extracytoplasmic proteins in *E. coli* (Chao and Vogel, 2016). Thus, CpxQ may analogously reduce synthesis of problematic proteins to combat envelope stress (Chao and Vogel, 2016). Another example is the *sodF* 3′UTR-derived sRNA in *Streptomyces coelicolor* (Kim et al., 2014). This sRNA (90-nt-long) is derived from the *sodF* 3′UTR and represses the expression of *sodN*, thereby shutting off the synthesis of Ni-SOD during nickel starvation (Kim et al., 2014). In addition, a 75 nt sRNA SorX, generated by RNase E cleavage of the 3′UTR of RSs2461 mRNA in *Rhodobacter sphaeroides*, represses the expression of *potA* (Peng et al., 2016). PotA is involved in the uptake of spermidine, which affects the sensitivity of *R. sphaeroides* to organic hydroperoxides. Hence, the 3′UTR-derived sRNA SorX can repress the import of spermidine to counteract oxidative stress (Peng et al., 2016). Altogether, these examples suggest that 3′UTRs can function as a sRNA reservoir that can post-transcriptionally regulate the expression of physically unlinked genes in response to changing environments (Miyakoshi et al., 2015). A remaining issue that needs to be addressed is whether the cleavage of mRNA during maturation of 3′UTR derived sRNAs affects the mRNA decay of its own mRNA.

## Exploitation of 3′UTR-Mediated Control of Gene Expression

Regulation of mRNA stability is a common mechanism used by bacteria to regulate gene expression. mRNA stability is strongly affected by the initial endonucleolytic cleavage of mRNA (Laalami et al., 2014). Thus, the addition of a regulatory sequence at the 3′UTRs could be used to artificially control gene expression. A ribozyme is usually composed of a 50–150 nt RNA motif with intrinsic RNA cleavage activity (Doherty and Doudna, 2000). Felletti et al. (2016) reported that the incorporation of a ligand-dependent ribozyme in the 3′UTR region induces the cleavage of the 3′UTRs in a ligand-dependent manner, which could be employed to control gene expression. In addition, 3′UTRs engineering has been used to improve the soluble expression of heterologous enzymes, and thus can be used to fine-tune enzyme activity in microbial cells (Song et al., 2016). The above studies suggest that 3′UTRs could serve as targets for post-transcriptional regulation of gene expression, a feature that could be exploited by biotechnology, synthetic biology, and metabolic engineering.

## Conclusion and Perspectives

It was traditionally believed that bacterial 3′UTRs mainly contain a transcriptional terminator. Recently, long 3′UTRs (>100 nt) were identified by transcriptomic analysis in many bacterial transcripts (Rasmussen et al., 2009; Toledo-Arana et al., 2009; ten Broeke-Smits et al., 2010; Ruiz de los Mozos et al., 2013). Since ca.40–50 nt is a sufficient length for a transcriptional terminator (Ruiz de los Mozos et al., 2013), additional regulatory elements are predicted to exist in these long 3′UTRs. Consistent with this hypothesis, 3′UTRs were found to regulate mRNA decay and translation and to act as sRNA targets or reservoirs for adaptation to various environmental changes, such as changes in temperature, pH, and nutrition availability (Opdyke et al., 2004; Kim et al., 2014; Peng et al., 2016; Chao and Vogel, 2016; Zhu et al., 2016). Although these 3′UTRs employ different mechanisms to regulate their own gene expression or targeted gene expression, one common feature is the regulation of gene transcription in a post-transcriptional manner.

A number of functional 3′UTRs have been identified. However, it is very likely that many more 3′UTRs function as post-transcriptional regulators, given that long 3′UTRs are widely present in bacteria. Furthermore, additional mechanisms of 3′UTR-regulated gene expression are expected to be identified; for example, RNA-binding proteins might bind to 3′UTRs to regulate gene expression. A group of mRNA termed ‘cutoRNAs’, which have long 3′UTRs that overlap with a downstream, convergently transcribed gene, has recently been reported (Moody et al., 2013). The regulatory function of these cutoRNAs remains unclear. Identification of new functional 3′UTRs and their regulatory mechanisms will lead to a better understanding of how bacteria use 3′UTRs as post-transcriptional regulators to respond to changes in the environment, and how such 3′UTRs regulation interacts with other transcriptional or post-transcriptional regulators. This knowledge will provide insights for new or improved biotechnological applications. Further, since many 3′UTRs play an important role in the regulation of virulence gene expression, interfering with the roles of these 3′UTRs might comprise an interesting alternative strategy for controlling bacterial pathogens.

## Author Contributions

All authors listed have made a substantial, direct and intellectual contribution to the work, and approved it for publication.

## Conflict of Interest Statement

The authors declare that the research was conducted in the absence of any commercial or financial relationships that could be construed as a potential conflict of interest.

## References

[B1] Agaisse H. Lereclus D. (1996). STAB-SD: a shine-dalgarno sequence in the 5′ untranslated region is a determinant of mRNA stability. *Mol. Microbiol.* 20 633–643. 10.1046/j.1365-2958.1996.5401046.x 8736542

[B2] Babitzke P. Baker C. S. Romeo T. (2009). Regulation of translation initiation by RNA binding proteins. *Annu. Rev. Microbiol.* 63 27–44. 10.1146/annurev.micro.091208.073514 19385727 PMC4682898

[B3] Balaban N. Novick R. P. (1995). Translation of RNAIII, the *Staphylococcus aureus* agr regulatory RNA molecule, can be activated by a 3′-end deletion. *FEMS Microbiol. Lett.* 133 155–161. 10.1016/0378-1097(95)00356-a 8566701

[B4] Barreau C. Paillard L. Osborne H. B. (2005). AU-rich elements and associated factors: are there unifying principles? *Nucleic Acids Res.* 33 7138–7150. 10.1093/nar/gki1012 16391004 PMC1325018

[B5] Breaker R. R. (2011). Prospects for riboswitch discovery and analysis. *Mol. Cell.* 43 867–879. 10.1016/j.molcel.2011.08.024 21925376 PMC4140403

[B6] Chao Y. Papenfort K. Reinhardt R. Sharma C. M. Vogel J. (2012). An atlas of Hfq-bound transcripts reveals 3′ UTRs as a genomic reservoir of regulatory small RNAs. *EMBO J.* 31 4005–4019. 10.1038/emboj.2012.229 22922465 PMC3474919

[B7] Chao Y. Vogel J. (2016). A 3′ UTR-derived small RNA provides the regulatory noncoding arm of the inner membrane stress response. *Mol. Cell* 61 352–363. 10.1016/j.molcel.2015.12.023 26805574

[B8] Chen L. H. Emory S. A. Bricker A. L. Bouvet P. Belasco J. G. (1991). Structure and function of a bacterial mRNA stabilizer: analysis of the 5′ untranslated region of ompA mRNA. *J. Bacteriol.* 173 4578–4586. 10.1128/jb.173.15.4578-4586.1991 1713205 PMC208132

[B9] Danese P. N. Silhavy T. J. (1998). CpxP, a stress-combative member of the Cpx regulon. *J. Bacteriol.* 180 831–839. 9473036 10.1128/jb.180.4.831-839.1998 PMC106961

[B10] De Lay N. Schu D. J. Gottesman S. (2013). Bacterial small RNA-based negative regulation: Hfq and its accomplices. *J. Biol. Chem.* 288 7996–8003. 10.1074/jbc.R112.441386 23362267 PMC3605619

[B11] Deneke C. Lipowsky R. Valleriani A. (2013). Effect of ribosome shielding on mRNA stability. *Phys. Biol.* 10:046008 10.1088/1478-3975/10/4/046008 23883670

[B12] Doherty E. A. Doudna J. A. (2000). Ribozyme structures and mechanisms. *Annu. Rev. Biochem.* 69 597–615. 10.1146/annurev.biochem.69.1.597 10966470

[B13] Felden B. Vandenesch F. Bouloc P. Romby P. (2011). The *Staphylococcus aureus* RNome and its commitment to virulence. *PLoS Pathog.* 7:e1002006 10.1371/journal.ppat.1002006 PMC3053349 21423670

[B14] Felletti M. Bieber A. Hartig J. S. (2016). The 3′-untranslated region of mRNAs as a site for ribozyme cleavage-dependent processing and control in bacteria. *RNA Biol.* 10.1080/15476286.2016.1240141 [Epub ahead of print]. PMC5785228 27690736

[B15] Geissmann T. Marzi S. Romby P. (2009). The role of mRNA structure in translational control in bacteria. *RNA Biol.* 6 153–160. 10.4161/rna.6.2.8047 19885993

[B16] Gerstmeir R. Wendisch V. F. Schnicke S. Ruan H. Farwick M. Reinscheid D. (2003). Acetate metabolism and its regulation in *Corynebacterium glutamicum*. *J. Biotechnol.* 104 99–122. 10.1016/S0168-1656(03)00167-6 12948633

[B17] Gossringer M. Hartmann R. K. (2012). 3′-UTRs as a source of regulatory RNAs in bacteria. *EMBO J.* 31 3958–3960. 10.1038/emboj.2012.269 23010777 PMC3474932

[B18] Henkin T. M. (2008). Riboswitch RNAs: using RNA to sense cellular metabolism. *Genes Dev.* 22 3383–3390. 10.1101/gad.1747308 19141470 PMC3959987

[B19] Hinnebusch A. G. Ivanov I. P. Sonenberg N. (2016). Translational control by 5′-untranslated regions of eukaryotic mRNAs. *Science* 352 1413–1416. 10.1126/science.aad9868 27313038 PMC7422601

[B20] Kim H. M. Shin J. H. Cho Y. B. Roe J. H. (2014). Inverse regulation of Fe- and Ni-containing SOD genes by a fur family regulator Nur through small RNA processed from 3′UTR of the sodF mRNA. *Nucleic Acids Res.* 42 2003–2014. 10.1093/nar/gkt1071 24234448 PMC3919588

[B21] Kirillina O. Fetherston J. D. Bobrov A. G. Abney J. Perry R. D. (2004). HmsP, a putative phosphodiesterase, and HmsT, a putative diguanylate cyclase, control Hms-dependent biofilm formation in *Yersinia pestis*. *Mol. Microbiol.* 54 75–88. 10.1111/j.1365-2958.2004.04253.x 15458406

[B22] Kortmann J. Narberhaus F. (2012). Bacterial RNA thermometers: molecular zippers and switches. *Nat. Rev. Microbiol.* 10 255–265. 10.1038/nrmicro2730 22421878

[B23] Krajewski S. S. Narberhaus F. (2014). Temperature-driven differential gene expression by RNA thermosensors. *Biochim. Biophys. Acta* 1839 978–988. 10.1016/j.bbagrm.2014.03.006 24657524

[B24] Laalami S. Zig L. Putzer H. (2014). Initiation of mRNA decay in bacteria. *Cell Mol. Life. Sci.* 71 1799–1828. 10.1007/s00018-013-1472-4 24064983 PMC3997798

[B25] Lalaouna D. Simoneau-Roy M. Lafontaine D. Masse E. (2013). Regulatory RNAs and target mRNA decay in prokaryotes. *Biochim. Biophys. Acta* 1829 742–747. 10.1016/j.bbagrm.2013.02.013 23500183

[B26] Lasa I. Toledo-Arana A. Dobin A. Villanueva M. de los Mozos I. R. Vergara-Irigaray M. (2011). Genome-wide antisense transcription drives mRNA processing in bacteria. *Proc. Natl. Acad. Sci. U.S.A.* 108 20172–20177. 10.1073/pnas.1113521108 22123973 PMC3250193

[B27] Liu B. Kearns D. B. Bechhofer D. H. (2016). Expression of multiple *Bacillus subtilis* genes is controlled by decay of slrA mRNA from Rho-dependent 3′ ends. *Nucleic Acids Res.* 44 3364–3372. 10.1093/nar/gkw069 26857544 PMC4838369

[B28] Lopez-Garrido J. Puerta-Fernandez E. Casadesus J. (2014). A eukaryotic-like 3′ untranslated region in *Salmonella enterica* hilD mRNA. *Nucleic Acids Res.* 42 5894–5906. 10.1093/nar/gku222 24682814 PMC4027200

[B29] Maeda T. Wachi M. (2012). 3′ Untranslated region-dependent degradation of the aceA mRNA, encoding the glyoxylate cycle enzyme isocitrate lyase, by RNase E/G in *Corynebacterium glutamicum*. *Appl. Environ. Microbiol.* 78 8753–8761. 10.1128/AEM.02304-12 23042181 PMC3502937

[B30] Mazumder B. Seshadri V. Fox P. L. (2003). Translational control by the 3′-UTR: the ends specify the means. *Trends Biochem. Sci.* 28 91–98. 10.1016/S0968-0004(03)00002-1 12575997

[B31] Miyakoshi M. Chao Y. Vogel J. (2015). Regulatory small RNAs from the 3′ regions of bacterial mRNAs. *Curr. Opin. Microbiol.* 24 132–139. 10.1016/j.mib.2015.01.013 25677420

[B32] Moody M. J. Young R. A. Jones S. E. Elliot M. A. (2013). Comparative analysis of non-coding RNAs in the antibiotic-producing Streptomyces bacteria. *BMC Genomics* 14:558 10.1186/1471-2164-14-558 PMC3765725 23947565

[B33] Nakamoto T. (2009). Evolution and the universality of the mechanism of initiation of protein synthesis. *Gene* 432 1–6. 10.1016/j.gene.2008.11.001 19056476

[B34] Novick R. P. Ross H. F. Projan S. J. Kornblum J. Kreiswirth B. Moghazeh S. (1993). Synthesis of staphylococcal virulence factors is controlled by a regulatory RNA molecule. *EMBO J.* 12 3967–3975. 7691599 10.1002/j.1460-2075.1993.tb06074.x PMC413679

[B35] Oliva G. Sahr T. Buchrieser C. (2015). Small RNAs, 5′ UTR elements and RNA-binding proteins in intracellular bacteria: impact on metabolism and virulence. *FEMS Microbiol. Rev.* 39 331–349. 10.1093/femsre/fuv022 26009640

[B36] Opdyke J. A. Kang J. G. Storz G. (2004). GadY, a small-RNA regulator of acid response genes in *Escherichia coli*. *J. Bacteriol.* 186 6698–6705. 10.1128/JB.186.20.6698-6705.2004 15466020 PMC522195

[B37] Peng T. Berghoff B. A. Oh J. I. Weber L. Schirmer J. Schwarz J. (2016). Regulation of a polyamine transporter by the conserved 3′ UTR-derived sRNA SorX confers resistance to singlet oxygen and organic hydroperoxides in *Rhodobacter sphaeroides*. *RNA Biol.* 13 988–999. 10.1080/15476286.2016.1212152 27420112 PMC5056773

[B38] Pesole G. Mignone F. Gissi C. Grillo G. Licciulli F. Liuni S. (2001). Structural and functional features of eukaryotic mRNA untranslated regions. *Gene* 276 73–81. 10.1016/S0378-1119(01)00674-6 11591473

[B39] Rasmussen S. Nielsen H. B. Jarmer H. (2009). The transcriptionally active regions in the genome of *Bacillus subtilis*. *Mol. Microbiol.* 73 1043–1057. 10.1111/j.1365-2958.2009.06830.x 19682248 PMC2784878

[B40] Ruiz de los Mozos I. Vergara-Irigaray M. Segura V. Villanueva M. Bitarte N. Saramago M. (2013). Base pairing interaction between 5′- and 3′-UTRs controls icaR mRNA translation in *Staphylococcus aureus*. *PLoS Genet.* 9:e1004001 10.1371/journal.pgen.1004001 PMC3868564 24367275

[B41] Selinger D. W. Saxena R. M. Cheung K. J. Church G. M. Rosenow C. (2003). Global RNA half-life analysis in *Escherichia coli* reveals positional patterns of transcript degradation. *Genome Res.* 13 216–223. 10.1101/gr.912603 12566399 PMC420366

[B42] Serganov A. Patel D. J. (2012). Metabolite recognition principles and molecular mechanisms underlying riboswitch function. *Annu. Rev. Biophys.* 41 343–370. 10.1146/annurev-biophys-101211-113224 22577823 PMC4696762

[B43] Silvaggi J. M. Perkins J. B. Losick R. (2005). Small untranslated RNA antitoxin in *Bacillus subtilis*. *J. Bacteriol.* 187 6641–6650. 10.1128/JB.187.19.6641-6650.2005 16166525 PMC1251590

[B44] Sonenberg N. (1994). mRNA translation: influence of the 5′ and 3′ untranslated regions. *Curr. Opin. Genet. Dev* 4 310–315. 10.1016/S0959-437X(05)80059-0 8032210

[B45] Song J. W. Woo J. M. Jung G. Y. Bornscheuer U. T. Park J. B. (2016). 3′-UTR engineering to improve soluble expression and fine-tuning of activity of cascade enzymes in *Escherichia coli*. *Sci. Rep.* 6:29406 10.1038/srep29406 PMC4942690 27406241

[B46] St Johnston D. (1995). The intracellular localization of messenger RNAs. *Cell* 81 161–170. 10.1016/0092-8674(95)90324-0 7736568

[B47] ten Broeke-Smits N. J. Pronk T. E. Jongerius I. Bruning O. Wittink F. R. Breit T. M. (2010). Operon structure of *Staphylococcus aureus*. *Nucleic Acids Res.* 38 3263–3274. 10.1093/nar/gkq058 20150412 PMC2879529

[B48] Thisted T. Sorensen N. S. Gerdes K. (1995). Mechanism of post-segregational killing: secondary structure analysis of the entire Hok mRNA from plasmid R1 suggests a fold-back structure that prevents translation and antisense RNA binding. *J. Mol. Biol.* 247 859–873. 10.1006/jmbi.1995.0186 7536849

[B49] Toledo-Arana A. Dussurget O. Nikitas G. Sesto N. Guet-Revillet H. Balestrino D. (2009). The Listeria transcriptional landscape from saprophytism to virulence. *Nature* 459 950–956. 10.1038/nature08080 19448609

[B50] Tree J. J. Granneman S. McAteer S. P. Tollervey D. Gally D. L. (2014). Identification of bacteriophage-encoded anti-sRNAs in pathogenic *Escherichia coli*. *Mol. Cell* 55 199–213. 10.1016/j.molcel.2014.05.006 24910100 PMC4104026

[B51] Van Assche E. Van Puyvelde S. Vanderleyden J. Steenackers H. P. (2015). RNA-binding proteins involved in post-transcriptional regulation in bacteria. *Front. Microbiol.* 6:141 10.3389/fmicb.2015.00141 PMC4347634 25784899

[B52] Wagner E. G. (2013). Cycling of RNAs on Hfq. *RNA Biol.* 10 619–626. 10.4161/rna.24044 23466677 PMC3710369

[B53] Waters L. S. Storz G. (2009). Regulatory RNAs in bacteria. *Cell* 136 615–628. 10.1016/j.cell.2009.01.043 19239884 PMC3132550

[B54] Wilkie G. S. Dickson K. S. Gray N. K. (2003). Regulation of mRNA translation by 5′- and 3′-UTR-binding factors. *Trends Biochem. Sci.* 28 182–188. 10.1016/S0968-0004(03)00051-3 12713901

[B55] Zhu H. Mao X. J. Guo X. P. Sun Y. C. (2016). The hmsT 3′ untranslated region mediates c-di-GMP metabolism and biofilm formation in *Yersinia pestis*. *Mol. Microbiol.* 99 1167–1178. 10.1111/mmi.13301 26711808

